# Ecological differentiation and assembly processes of abundant and rare bacterial subcommunities in karst groundwater

**DOI:** 10.3389/fmicb.2023.1111383

**Published:** 2023-07-25

**Authors:** Sining Zhong, Bowen Hou, Jinzheng Zhang, Yichu Wang, Xuming Xu, Bin Li, Jinren Ni

**Affiliations:** ^1^Fujian Provincial Key Laboratory of Soil Environment Health and Regulation, College of Resources and Environment, Fujian Agriculture and Forestry University, Fuzhou, China; ^2^College of Environmental Sciences and Engineering, Peking University, Beijing, China; ^3^State Environmental Protection Key Laboratory of All Material Fluxes in River Ecosystems, College of Environmental Sciences and Engineering, Peking University, Beijing, China; ^4^State Key Laboratory of Eco-hydraulics in Northwest Arid Region of China, Xi'an University of Technology, Xi'an, China; ^5^College of Water Sciences, Beijing Normal University, Beijing, China

**Keywords:** bacterial community, abundant and rare taxa, karst groundwater, assembly processes, environmental thresholds

## Abstract

The ecological health of karst groundwater has been of global concern due to increasing anthropogenic activities. Bacteria comprising a few abundant taxa (AT) and plentiful rare taxa (RT) play essential roles in maintaining ecosystem stability, yet limited information is known about their ecological differentiation and assembly processes in karst groundwater. Based on a metabarcoding analysis of 64 groundwater samples from typical karst regions in southwest China, we revealed the environmental drivers, ecological roles, and assembly mechanisms of abundant and rare bacterial communities. We found a relatively high abundance of potential functional groups associated with parasites and pathogens in karst groundwater, which might be linked to the frequent regional anthropogenic activities. Our study confirmed that AT was dominated by Proteobacteria and Campilobacterota, while Patescibacteria and Chloroflexi flourished more in the RT subcommunity. The node-level topological features of the co-occurrence network indicated that AT might share similar niches and play more important roles in maintaining bacterial community stability. RT in karst groundwater was less environmentally constrained and showed a wider environmental threshold response to various environmental factors than AT. Deterministic processes, especially homogeneous selection, tended to be more important in the community assembly of AT, whereas the community assembly of RT was mainly controlled by stochastic processes. This study expanded our knowledge of the karst groundwater microbiome and was of great significance to the assessment of ecological stability and drinking water safety in karst regions.

## 1. Introduction

Karst landforms are broadly distributed in the world and represent 7–12% of the Earth's continental area (Zhang et al., 2018). More than 25% of the global population relies on karst groundwater for domestic drinking and irrigation purposes (Hartmann et al., 2014). Karst aquifers are characterized by unique features such as large voids, high flow velocities, and rapid infiltrations due to strong dissolution processes (White, 2002), leading to their high ecological sensitivity in response to climate changes and human activities (Ollivier et al., 2019; Olarinoye et al., 2020). Varieties of anthropogenic contaminants, such as pharmaceuticals, flame retardants, microplastics, and antibiotics (Reberski et al., 2022), have been widely detected in karstic aquifers. Due to the increasing pressure from these environmental issues, the ecological health and drinking water sustainability of karst groundwater has become a global concern (Tang et al., 2022).

Diversified microbes that colonize aquifers constitute the sole ecological community in groundwater ecosystems (Whitman et al., 1998; Magnabosco et al., 2018) and pivotally participate in multiple biogeochemical processes (e.g., carbon, nitrogen, sulfur, and phosphorus; Probst et al., 2018; Wang S. et al., 2019; Wang Y. et al., 2019). Bacterial communities are normally uneven in abundance and distribution, with a few species with high abundance (abundant taxa) and the majority with low abundance (rare taxa; Pedros-Alio, 2012; He et al., 2022; Zhao et al., 2022). Rare taxa, considered a crucial microbial “seed bank,” are ecological insurance for microbial diversity and community stability and provide disproportionately important functions (Shade et al., 2014; Liu et al., 2015a; Jiao and Lu, 2020). Generally, dominant taxa tend to exhibit strong environmental adaptation, but rare taxa would become dominant under suitable environmental conditions (Reddin et al., 2015; Kurm et al., 2019). The variety of “rare-to-prevalent” dynamics could be explained by the priority effects, awakening from dormancy, and heterogeneity of environmental preference (Lee et al., 2021; Zhang et al., 2022). Previous studies have documented the distinct spatial patterns and functional traits of abundant and rare bacteria in surface water such as rivers (Yi et al., 2022), lakes (Zhang et al., 2022), and oceans (Li et al., 2021). However, the biogeographic patterns and assembly mechanisms of abundant and rare bacterial subcommunities in groundwater remained unclear.

Rapid advances in sequencing and multi-omics technologies have made it possible to identify biogeographic patterns of bacterial diversity and structure at large scales, promoting our understanding of the ecological and evolutionary processes in natural ecosystems (Liu et al., 2018; Shi et al., 2018). Meta-analyses revealed that the substantially different bacterial communities between distinct habitats were driven by multiple environmental factors (e.g., salinity, nutrients, and heavy metals; Power et al., 2018; Carlson et al., 2019; Liu et al., 2020). Habitat specialization is usually the consequence of adaptive and metabolic evolution via natural selection (environmental filtering; Wang et al., 2013). Meanwhile, variation in bacterial communities is also influenced by stochastic processes (e.g., ecological drift, dispersal limitation, mass effects, and historical contingency; Bahram et al., 2016; Fodelianakis et al., 2017; Archer et al., 2019). To date, there is a consensus that both deterministic (niche-based) and stochastic (neutral) processes would simultaneously shape microbial community assembly but disentangling the balance between these two processes is still a complicated issue.

**Figure 1:**
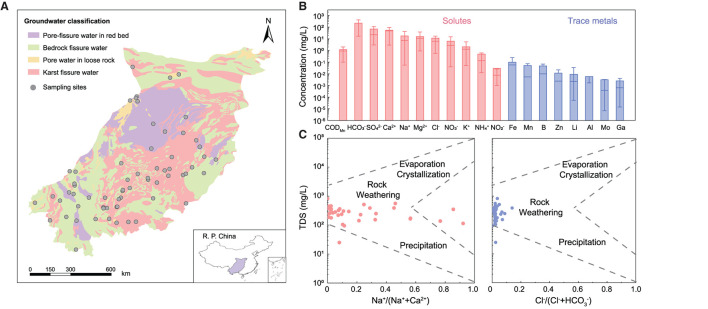
Physicochemical characteristics of karst groundwater in southwest China. **(A)** Map of typical karst areas showing the sampling sites in southwest China. **(B)** The content of main solutes and metals in karst groundwater. **(C)** Gibbs diagrams showing the dominant hydrochemical processes for karst groundwater.

Southwest China harbors the largest karst landscapes in the world and is one of the hotspots of global biodiversity (Li et al., 2022). As the main driver of groundwater ecosystems, the bacterial community affects the material and energy fluxes of the karst subterranean environments. Understanding the spatial variations, ecological drivers, and assembly processes of abundant and rare bacteria in karst groundwater is beneficial to assess its vulnerability and sustainable potential relevant to various human disturbances. In this study, we collected 64 groundwater samples from a karst area in southwest China (Figure 1A) and aimed to (a) reveal the bacterial diversity, structures, and potential functions of karst groundwater; (b) determine the composition and environmental adaptability of rare and abundant bacterial taxa; and (c) elucidate the ecological processes involved in shaping the abundant and rare subcommunities.

## 2. Materials and methods

### 2.1. Study area description and sampling

Southwest China (97°38′-113°40′ E, 21°03′-34°57′ N) has a subtropical/tropical humid monsoon climate with abundant annual rainfall ranging from 1,013 to 1,607 mm (Li et al., 2022). The continuous dissolution of widely distributed carbonate and sulfate rocks facilitates the development of stone forests and karst caves (Liu et al., 2021), thereby leading to the formation of the largest karst landform in the world (5.5 × 10^4^ km^2^). Considering typical topographic types, major rocky desertification zones, and potential ecologically fragile regions, we collected 64 groundwater samples from three provinces (i.e., Yunnan, Guizhou, and Guangxi) in southwest China during 2016–2017. Based on groundwater types identified by the China Geological Survey (https://geocloud.cgs.gov.cn/), these sampling sites included karst fissure water (42), pore-fissure water in red bed (5), bedrock fissure water (10), and pore water in loose rock (7), classified based on National Geological Survey (https://geocloud.cgs.gov.cn/). All groundwater samples were first-hand data from newly constructed wells according to the procedures of national standards (HJ/T 164-2004).

Before sample collection, groundwater was pumped out using a submersible sampling pump at a controlled discharge below 100 ml/min. Physicochemical properties (i.e., pH, conductivity, and oxidation–reduction values) of outflowing groundwater were measured with a portable tester for 15 min until three consecutive measurements were consistent (standard deviation < 5%). Following this purge, more than 3,000 L of groundwater were formally extracted and filtered by 0.01 μm hollow fiber membranes (Toray, Japan) to enrich microbial cells. All filtered membranes were immediately transported with dry ice to the designated laboratories. Then, the substances on the membranes were further extracted by ultrasonication, filtered by 0.22 μm polycarbonate membranes (Millipore, USA), and stored at −80°C before DNA extraction.

### 2.2. Groundwater physicochemical analysis

The longitude and latitude of each groundwater sample were recorded through a handheld GPS (Magellan, USA) during sampling. Groundwater samples for physicochemical analysis were collected in 5 L sterile bottles and transported to the laboratory at 4°C within 24 h. Standard methods were adopted to measure an array of physicochemical properties. The major metals [e.g., calcium (Ca), sodium (Na), magnesium (Mg), and potassium (K)] were measured by ICP-MS (Thermo Fisher Scientific, USA), while trace metals [e.g., manganese (Mn), iron (Fe), arsenic (As), and copper (Cu)] were determined by ICP-OES (Leeman, USA). The water samples were collected in 5 L sterile bottles for further analysis of total organic carbon (TOC; HJ 501–2009), ammonia nitrogen (NH_4_-N; HJ 535–2009), nitrate nitrogen (NO_3_-N; HJ/T 346–2007), and nitrite nitrogen (NO_2_-N; GB 7493–87).

### 2.3. DNA extraction and sequencing

The genomic DNA of each groundwater sample was extracted using the MoBio PowerSoil^®^ Kit (MoBio Laboratories, Carlsbad, CA, USA), according to the manufacturer's protocols. The DNA concentration and quality of mixed duplicate extracts were evaluated by a NanoDrop Spectrophotometer (NanoDrop Technologies Inc., Wilmington, DE, USA). The barcoded primer pairs 338F (ACTCCTACGGGAGG-CAGCAG) and 806R (GGACTACHVGGGTWTCTAAT-3) were used to amplify the bacterial 16S rRNA genes (Caporaso et al., 2011). Based on the concentration to generate amplicon libraries, PCR products were mixed in equal amounts and sequenced on the Illumina MiSeq PE300 platform (Illumina, San Diego, USA) at Shanghai Majorbio Bio-Pharm Technology Company Co., Ltd. (Shanghai, China).

### 2.4. Bioinformatics and statistical analyses

Bioinformatic analysis of the next-generation DNA sequencing data was performed using QIIME on the Majorbio cloud platform (https://cloud.majorbio.com/). Operational taxonomic units (OTUs) were clustered with 97% identity using UPARSE (Edgar, 2013), and chimeric sequences were removed using UCHIME. Bacterial OTUs were assigned using the RDP classifier (Wang et al., 2007) against the SILVA 16S rRNA database (http://www.arb-silva.de/). Bacteria were classified into different metabolic functional groups based on the FAPROTAX database (Louca et al., 2016; Sansupa et al., 2021). To account for the uneven sequencing depth among samples, the OTU table for subsequent comparative analysis was rarefied to the same sequencing depth, according to the minimum read count. We defined the abundant taxa as the average relative abundance of OTUs > 0.1%, the rare taxa as the relative abundance of < 0.01%, and the intermediate OTUs as the relative abundance between 0.01 and 0.1% (Jiao and Lu, 2020). All the raw sequencing datasets in this study have been deposited in the NCBI Sequence Read Archive, under accession number PRJNA692269.

Non-metric multidimensional scaling (NMDS) was used to visualize the dissimilarity of bacterial communities based on the Bray–Curtis distance, and similarity analysis (ANOSIM) was calculated to test the significance of differences in community structures. Variance partitioning analysis (VPA) was performed using pairwise Bray–Curtis dissimilarity to quantify the relative contribution of environmental and geographic factors, in addition to their combined effect on the spatial turnover of bacterial communities. A constrained correspondence analysis (CCA) with environmental variables was performed to interpret community distribution. NMDS, ANOSIM, CCA, and VPA were performed with the *vegan* packages in R software (https://www.r-project.org/). Mantel tests were used to evaluate the significance of Spearman's rank correlations between the Bray–Curtis dissimilarity and geographic distance matrices. The differences in bacterial composition were examined at *p* < 0.05, with Wilcoxon rank-sum tests. To calculate the threshold values of rare and abundant subcommunities responding to various environmental factors, we applied threshold indicator taxa analysis (TITAN) using the *TITAN2* R package (Jiao and Lu, 2020).

Pairwise Spearman's correlation coefficients based on the relative abundances were calculated only between OTUs that occurred in > 20% of samples. Then, the robust (Spearman's *r* > 0.60) and significant correlations (*p* < 0.01) were selected to filter the data for reduced network complexity. The network visualization and modular analyses were conducted with the interactive platform Gephi (version 0.9.2), and node-level topological features (i.e., degree, betweenness, and closeness centrality) were characterized with the igraph package in R software. The topological roles of nodes in the networks were classified by the threshold values of Zi (within-module connectivity) and Pi (among-module connectivity; Guimera and Amaral, 2005). Node attributes can be divided into four types, namely, module hubs (Zi > 2.5), network hubs (Zi > 2.5 and Pi > 0.62), connectors (Pi > 0.62), and peripherals (Zi < 2.5 and Pi < 0.62).

The neutral model of Sloan et al. was used to estimate the potential role of neutral processes in shaping microbial community structure by describing the relationship between the observed frequency of occurrence and the abundance of OTUs (Sloan et al., 2007). To quantify the relative importance of deterministic and stochastic processes in microbial community assembly, the normalized stochasticity ratio (NST) was estimated using the “tNST” and “nst.boot” functions with the NST package in R software, with 50% taken as the boundary point between more deterministic (< 50%) and more stochastic (>50%) assemblies (Guo et al., 2018; Ning et al., 2019). The null model framework was calculated with the picante and vegan packages in R software to estimate the relative importance of five ecological processes (i.e., homogenizing selection, variable selection, dispersal limitation, homogenizing dispersal, and ecological drift) in bacterial community assembly (Stegen et al., 2013).

## 3. Results

### 3.1. Physicochemical characteristics of karst groundwater in southwest China

Most groundwater samples (85.0%) were weakly alkaline with an average pH of 7.51 (6.31–11.35), which was mainly related to chemical erosion of carbonate minerals and accumulation of ${\text{HCO}}_{3}^{-}$ in the study area (average 232.97 mg/L; Li et al., 2018). According to the standard for Groundwater Quality of China (GB/T 14848–2017), 78.1–100% of groundwater samples were satisfied with a fairly good level (I, II, or III). ${\text{NO}}_{3}^{-}$-N, ${\text{NH}}_{4}^{+}$-N, F^−^, and Mn could be identified as potential threats to groundwater quality, with >10% of samples at the poor level (IV or V; Figure 1B and Supplementary Table 1). High concentrations of ${\text{NO}}_{3}^{-}$-N (average 13.2 mg/L) and ${\text{NH}}_{4}^{+}$-N (average 0.45 mg/L) in karst groundwater were potentially attributed to anthropogenic loadings considering the high permeability of karst rocks (Opsahl et al., 2017) and extensive agricultural fertilization in southwest China (Zhang, 2020). Fe (0.13 mg/L), B (0.07 mg/L), and Mn (0.05 mg/L) were the dominant trace metals in karst groundwater, which are closely related to the regional geological environments (Peng et al., 2022).

The concentration of total dissolved solids (TDS) ranged from 25 to ~1.792 mg/L with an average value of 361.79 mg/L, indicating low salinity in karat groundwater. Ca^2+^ (45.99 mg/L), Na^+^ (26.93 mg/L), and Mg^2+^ (12.77 mg/L) were identified as major cations while ${\text{HCO}}_{3}^{-}$ (232.97 mg/L) and ${\text{SO}}_{4}^{2-}$ (97.31 mg/L) were dominant anions. Piper analysis further showed that the hydrochemical types of the groundwater samples were assigned to Mg^2+^-Ca^2+^-${\text{HCO}}_{3}^{-}$ category, which suggested a remarkable leaching process in karst regions (Supplementary Figure 1). Gibbs diagrams indicated that the chemical evolution of most groundwater samples (93.5%) was mainly controlled by rock weathering (Figure 1C), reflecting the chronological impacts of soluble rocks on karst groundwater (Li et al., 2018).

### 3.2. Diversity, structure, and potential function of the bacterial community

After quality filtering and the removal of chimeric sequences, high-throughput Illumina sequencing yielded a total of 24,321 OTUs from 64 karst groundwater samples in southwest China. The rarefaction curves (Supplementary Figure 2) and high Good's coverage values (0.973 ± 0.002, Supplementary Table 2) of each sample illustrated that the majority of bacterial taxa were well-covered by the current sequencing depth. The bacterial alpha diversity in karst groundwater, including Chao1, Shannon diversity, and phylogenetic diversity (pd), exhibited a more significant relationship with geographic factors (i.e., longitude, latitude, and well depth) than with environmental variables (Supplementary Figure 3).

Among the 67 identified phyla, Proteobacteria was the most dominant phylum, accounting for 21.55% of the total OTUs and 63.5% of the total sequences, followed by Campylobacterota, Bacteroidota, Actinobacteriota, Patescibacteria, Firmicutes, and Desulfobacterota (Supplementary Figure 4). As the most concerned superphylum in recent years (Herrmann et al., 2019; Chaudhari et al., 2021; Ruiz-Gonzalez et al., 2022), Patescibacteria had abundant phylotypes in karst groundwater (18.7% of the total OTUs), second only to Proteobacteria. The NMDS and ANOSIM analyses illustrated the significant discrepancy in bacterial composition (Supplementary Figure 5A) between phreatic and confined water, classified based on the burial condition of the karst region. Community similarity among samples in phreatic water was much higher than that in confined water (Wilcoxon rank-sum test: *p* < 0.01). At the genus level, *Aquabacterium, Acidovorax, Cavicella, Simplicispira*, and *Polaromonas* all belonging to Proteobacteria, preferred the phreatic water, whereas *Acetobacterium* (Firmicutes), *Perlucidibaca* (Proteobacteria), and *Desulfovibrio* (Desulfobacterota) were relatively abundant in confined water (Supplementary Figure 6). Moreover, CCA results suggested that longitude, well depth, and some geochemical variables (e.g., K^+^, Mg^2+^, and ${\text{HCO}}_{3}^{-}$) significantly impacted the spatial patterns of bacterial communities in karst groundwater (Supplementary Figure 5B).

**Figure 2:**
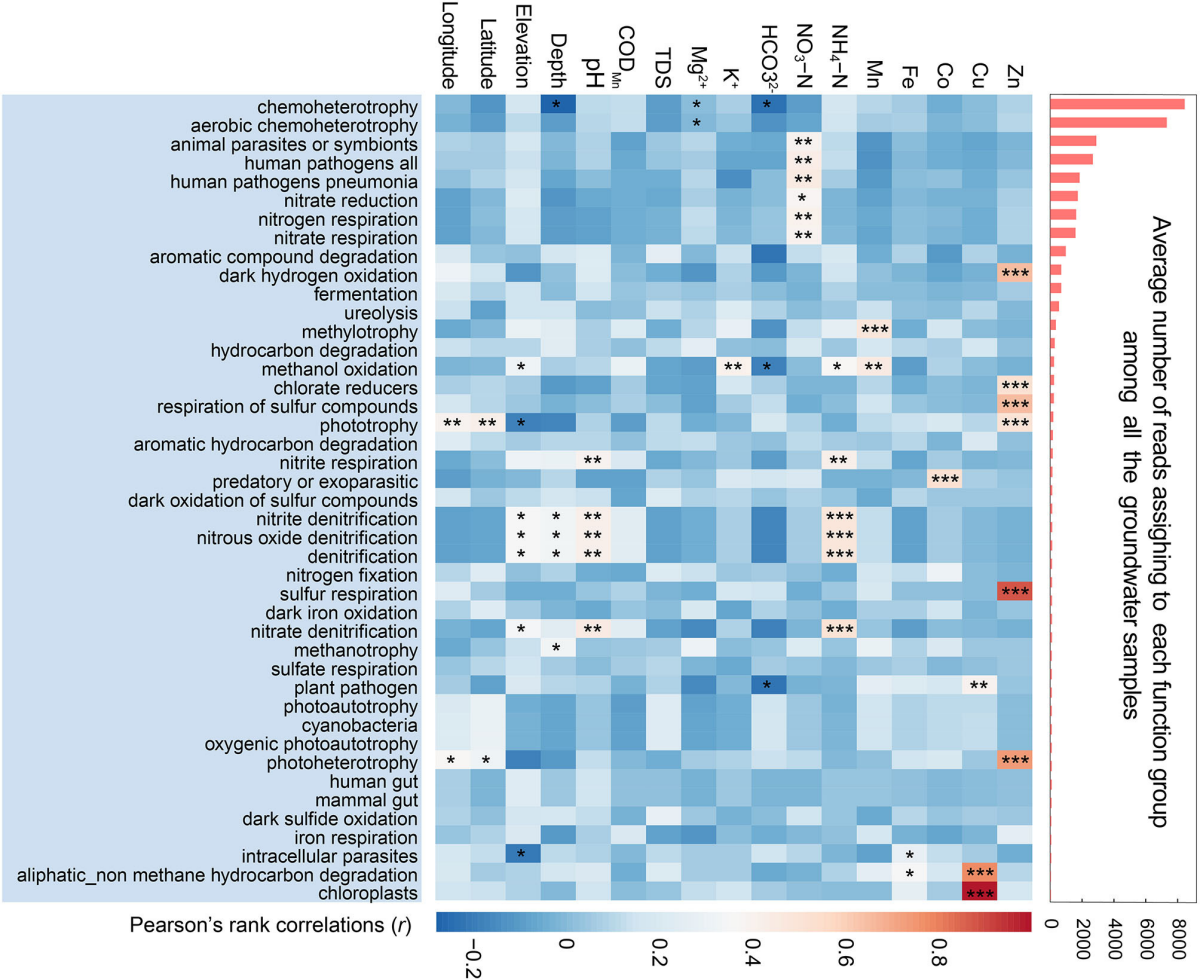
Bacterial functional profiles in karst groundwater. The bar chart on the right indicates the average number of reads assigned to each functional group predicted by FAPROTAX. The heatmap on the left shows Spearman's correlations between the abundances of functional groups and environmental variables. The asterisks denoted the significance of statistical tests: ***p < 0.001, **0.001 < *p* < 0.01, and *0.01 < *p* < 0.05.

The results of potential function predicted by FAPROTAX (Figure 2) showed that chemoheterotrophy, especially aerobic chemoheterotrophy, was the most abundant functional group even in such oligotrophic and anoxic subterranean environments, negatively associated with well depth and ${\text{HCO}}_{3}^{-}$. The relatively high abundance of potential functional groups associated with animal parasites and human pathogens in karst groundwater was strongly and positively correlated with the content of NO_3_-N. In terms of the functional groups relevant to nitrogenous compound cycles, nitrate reduction, nitrogen respiration, and nitrate respiration were correlated with NO_3_-N, while nitrite denitrification, nitrous oxide denitrification, and denitrification were strongly associated with NH_4_-N.

### 3.3. Rare/abundant subcommunities and their environmental responses

**Figure 3:**
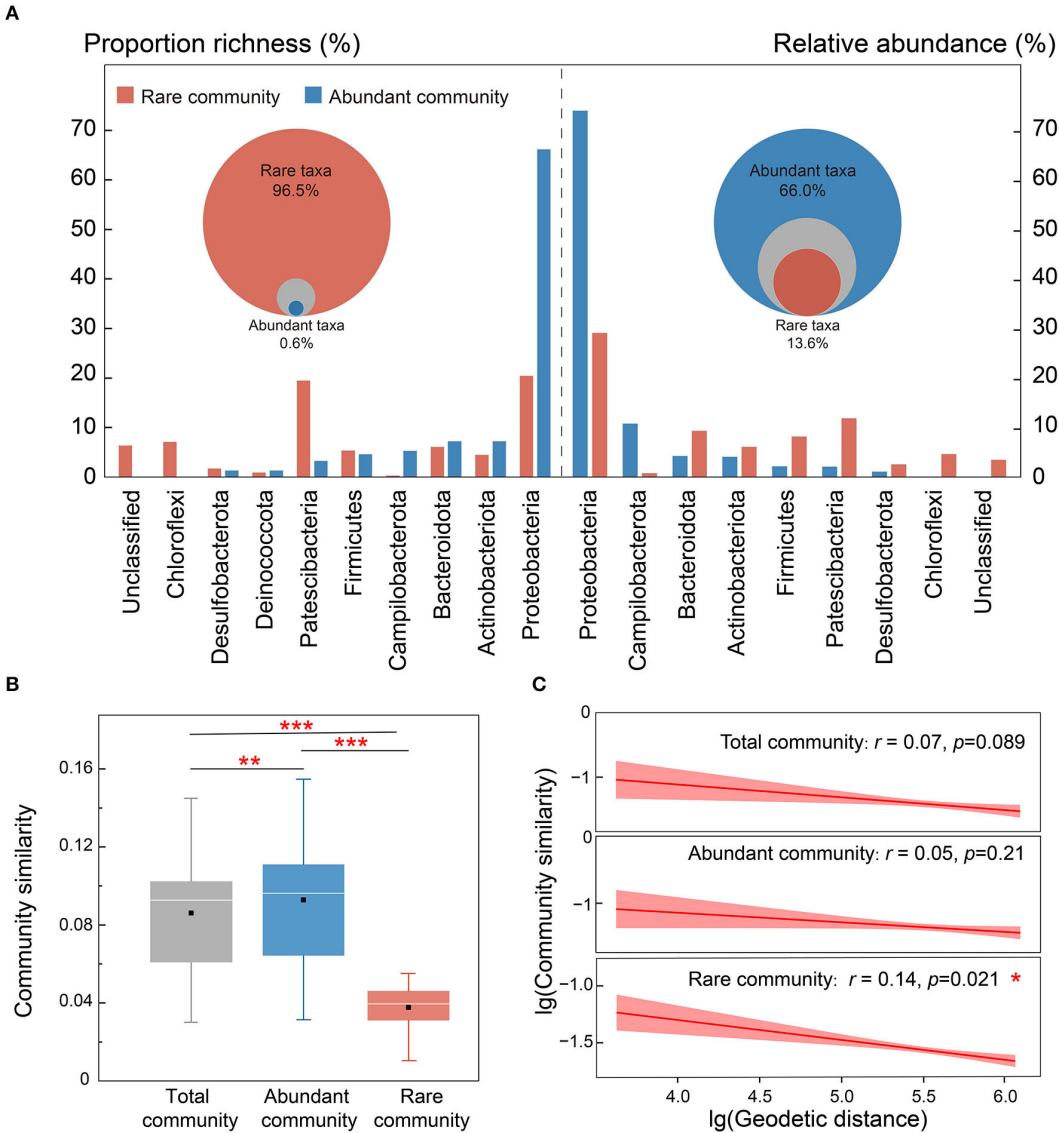
The compositional differentiation of rare and abundant subcommunities in karst groundwater. **(A)** Relative abundances of the major phyla in rare and abundant subcommunities. Only the phyla with mean proportional richness or relative abundances of > 1% are shown. Bacterial compositional differences. **(B)** The discrepancy in community similarity between total and abundant taxa in karst groundwater. **(C)** Geodetic distance–decay curves of total and abundant communities based on Bray–Curtis similarities. The colored solid lines indicate the ordinary least squares linear regressions, with the shaded areas representing 95% confidence intervals. Mantel Spearman's *r* and *p*-values are stated. The asterisks denote the significance of statistical tests: ^***^p < 0.001, ^**^0.001 < *p* < 0.01, and ^*^0.01 < *p* < 0.05.

Only 151 OTUs (0.62% of total OTUs) were identified as abundant taxa (AT), yet they accounted for 66.0% of the average relative abundance. Conversely, rare taxa (RT) accounted for more than 96.5% of the total OTUs with an average relative abundance of merely 13.6% (Figure 3A). Proteobacteria was the most abundant phylum in both abundant and rare subcommunities, though the proportion richness and relative abundance of AT (73.9 and 66.2% of the sequence and OTUs, separately) were much higher than those of RT (29.0 and 20.0%, *p* < 0.05). Most members of Campilobacterota were identified as AT (5.2%) rather than RT (0.3%), while Patescibacteria and Chloroflexi flourished more in RT (26.6%) than in AT (3.3%).

**Figure 4:**
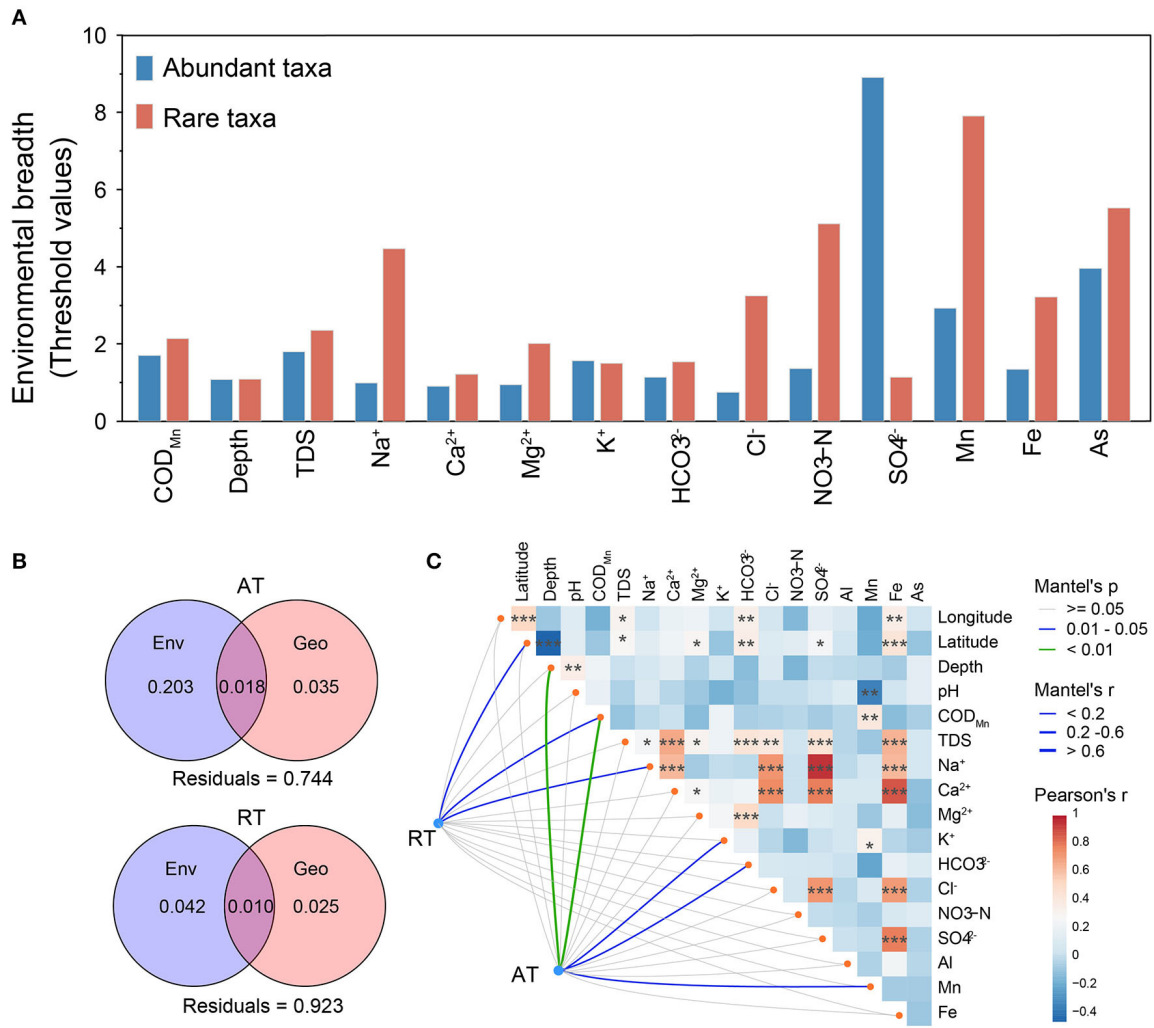
Environmental drivers of abundant and rare subcommunities in karst groundwater. **(A)** The environmental breadth of groundwater physicochemical variables for abundant and rare subcommunities is estimated through threshold indicator taxon analyses (TITAN). The threshold value ranges were standardized to non-dimensionalize selected physicochemical variables. **(B)** Variance partition analysis shows the relative contributions of environmental (Env.) and geographic (Geo.) factors and their combined effect on community variations. **(C)** The response of the rare and abundant subcommunities to environmental variables based on Mantel tests. The edge color indicates Mantel's *p*-value, and the width of the edges indicates Mantel's *r*-value. Pearson's correlation coefficients between environmental variables are displayed with the color gradient. All the asterisks denote the significance of statistical tests: ***p < 0.001, **0.001 < *p* < 0.01, and *0.01 < *p* < 0.05.

As shown in Figure 3B, the community similarity of AT was significantly higher than that of total taxa (Wilcoxon rank-sum test, *p* < 0.01) and RT (*p* < 0.001), suggesting that rare taxa were the main driver of community differences in karst groundwater. The rare subcommunity showed a significant distance-decay relationship (DDR), in which community similarity decreased with increasing geographic distance (Wu et al., 2019), while no significant DDR was observed for the abundant subcommunity (Figure 3C), indicating that AT was less constrained by geographic distance in karst groundwater. The rare subcommunity exhibited broader environmental threshold ranges in response to most environmental variables than the abundant subcommunity, based on the TITAN2 analysis (Figure 4A and Supplementary Figure 7). Notably, the environmental threshold ranges of Na^+^ (Valuerare = 4.47; Valueabundant = 1.0) and Cl^−^ (Valuerare = 3.2; Valueabundant =0.75) for the rare subcommunity were most significantly higher than those of the abundant subcommunity, whereas only those of ${\text{SO}}_{4}^{2-}$ were higher for the abundant subcommunity (Valuerare = 1.1; Valueabundant = 8.9). As the results of VPA (Figure 4B), 3.5%, 1.8%, and 20.3% of the variation of the abundant subcommunity were explained by geographic distance, environmental variables, and their interactions respectively, and the proportions of explaining were much higher than that of the rare subcommunity (2.5, 1.0, and 4.2%, respectively). Mantel and CCA results further confirmed that the structures of rare subcommunity were significantly affected by geographic factors (i.e., latitude and longitude) rather than hydrochemical factors, while well depth, K^+^, ${\text{HCO}}_{3}^{-}$, NO_3_-N, COD_Mn_, and some metals (e.g., Mn and As) impacted the spatial distribution of abundant subcommunity in karst groundwater (Figure 4C and Supplementary Figure 8).

### 3.4. Co-occurrence network of bacterial communities in karst groundwater

**Figure 5:**
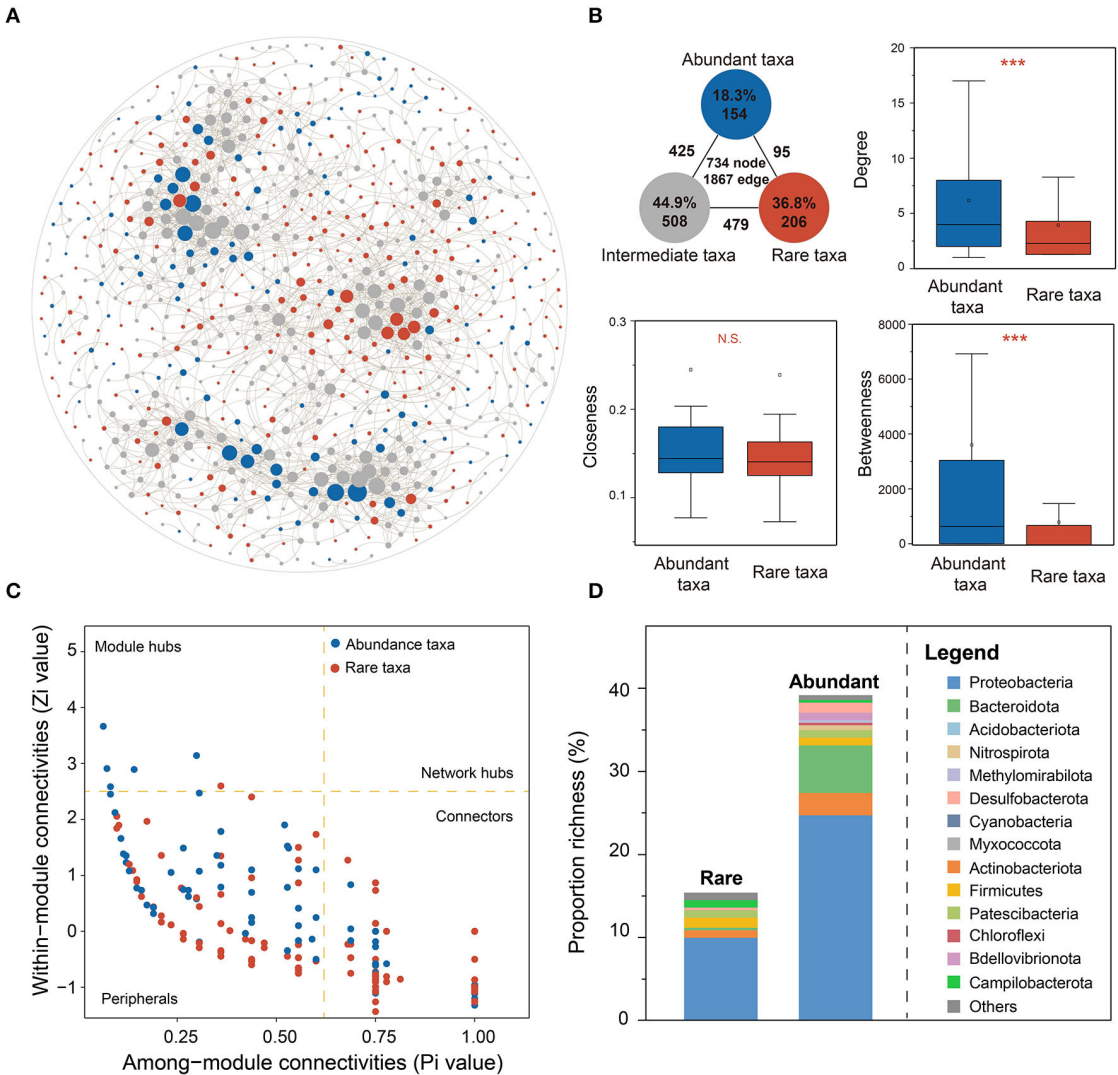
Ecological roles of AT and RT in the bacterial co-occurrence network. **(A)** The bacterial co-occurrence network was constructed based on a strong (Spearman's *r* > 0.6) and significant (FDR-adjusted *p* < 0.05) correlation among total OTUs. **(B)** The node-level topological features of AT and RT include degree, betweenness centrality, and closeness centrality. **(C)** Network roles of AT and RT based on the values of Zi and Pi. **(D)** The composition of keystone species for AT and RT. The asterisks denote the significance of statistical tests: ^***^p < 0.001, ^**^0.001 < *p* < 0.01, and ^*^0.01 < *p* < 0.05.

A bacterial co-occurrence network was constructed based on Spearman's correlations among total OTUs (|*r*| > 0.6 and FDR-adjusted *p* < 0.01, Figure 5A) to reveal the ecological roles and interrelationships of abundant and rare taxa in karst groundwater. The network degree of the bacterial community was distributed according to the power-law distribution pattern, which suggested that the co-occurrence network was reliable, scale-free, and non-random (Bergman and Siegal, 2003; Steele et al., 2011; Barberán et al., 2012). Intermediate OTUs dominated bacterial co-occurrence networks (44.9% of nodes) and connected more closely than rare and abundant taxa (75.6% of edges). The inter-connections between rare and abundant taxa only accounted for 5.1% of total edges, suggesting poor rare-abundant interactions in karst groundwater. AT exhibited stronger inter-connectivity than RT, characterized by a higher average degree of betweenness and clustering coefficient (Figure 5B), which indicated that AT might share similar niches and play more important roles in maintaining bacterial community stability in karst groundwater.

Based on the values of Zi and Pi, the network hub (Zi > 2.5 and Pi > 0.62), module hub (Zi > 2.5), and connector (Pi > 0.62) could be identified as the keystone taxa of the co-occurrence network (Figure 5C and Supplementary Table 3). Of 15 module hubs and 332 connectors, more RTs were identified as connectors, while more module hubs were ATs than RTs. The keystone taxa of AT were primarily composed of Proteobacteria (33), Acidobacteriota (4), Patescibacteria (3), and Campilobacterota (3) (Figure 5D), while those of RT mostly belonged to Proteobacteria (82), Bacteroidota (19), Actinobacteriota (9), Desulfobacterota (4), Firmicutes (3), Patescibacteria (3), and Bdellovibrionota (3). Furthermore, we explored how environmental variables influence community stability by unraveling the responses of module hubs to environmental changes (Supplementary Figure 9). As a result, the module hubs of AT exhibited similar responses to environmental variation, which were strongly correlated with well depth, total hardness, TDS, Na^+^, Ca^2+^, Cl^−^, and Fe, while module hubs belonging to rare and intermediate taxa were little affected by environmental factors.

### 3.5. Assembly processes of abundant and rare subcommunities

**Figure 6:**
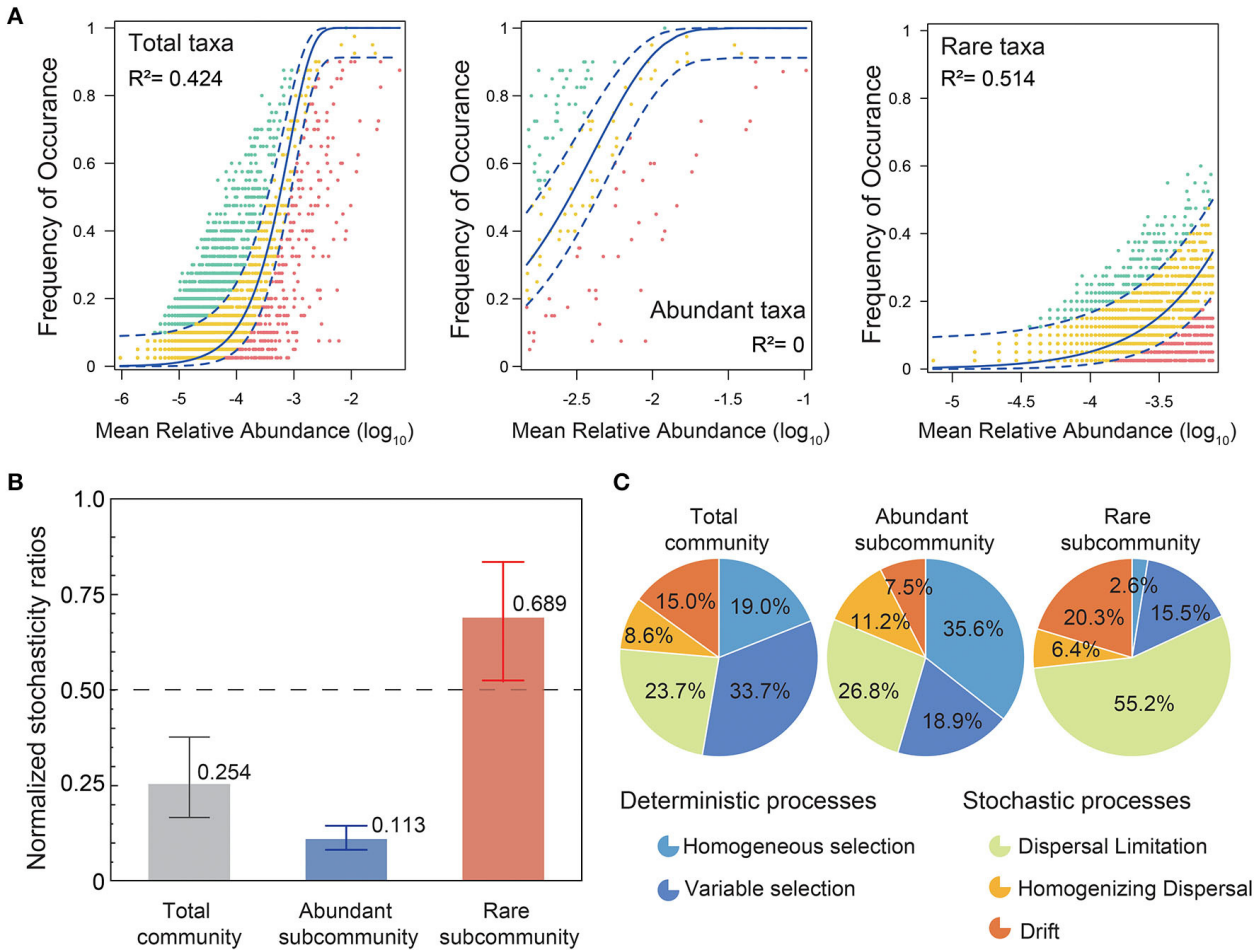
Assembly processes of abundant and rare subcommunities. **(A)** The neutral interpretations of total, abundant, and rare subcommunities are based on the neutral community model (NCM). *R*^2^ denotes the fitting goodness of the neutral model. Green circles indicate that OTUs occur more frequently than predicted by the NCM, while red circles represent those that occur less frequently than predicted. The solid blue line shows the best-fitting neutral models and the dashed lines depict the 95% confidence intervals for the models. **(B)** Mean values of normalized stochasticity ratios (NST) of total, abundant, and rare subcommunities, with 0.5 taken as the boundary point between more deterministic (<0.5) and more stochastic (>0.5) assemblies. **(C)** Null model analysis of deterministic and stochastic processes in the community assemblies of total, abundant, and rare subcommunities. Deterministic processes include variable selection and homogeneous selection, while stochastic processes include dispersal limitation, homogenizing dispersal, and drift.

To investigate the underlying mechanisms of coexistence and spatial distribution of bacterial communities in karst groundwater, we assessed the contributions of deterministic and stochastic processes in the community assembly of abundant and rare taxa by the ecological models (Figure 6). The neutral interpretation of the bacterial community in karst groundwater (*R*^2^ = 0.424) was much lower than in other natural surface ecosystems (Burns et al., 2016; Liu et al., 2018), which indicated that bacteria in karst groundwater suffered a stronger dispersal limitation than in surface ecosystems. The neutral interpretation (*R*^2^ = 0.514) of rare subcommunities was much higher than that of abundant subcommunities. Based on NST, the total community, especially the abundant subcommunity, was mainly governed by deterministic processes (total: 0.254, abundance: 0.113), while the rare subcommunity was more regulated by stochasticity (mean NSTs = 0.689; Figure 5B). Furthermore, the null model analysis suggested that deterministic processes (54.5%) prevailed primarily in abundant subcommunity assemblies, while the majority of rare taxa (81.9%) were controlled by stochastic processes (Figure 5C). Particularly, dispersal limitation contributed a larger proportion to the assembly of rare subcommunities (55.2%) compared with abundant ones (26.8%), while homogeneous selection influenced the abundant taxa (35.6%) more than the rare taxa (2.6%). Homogenizing dispersal had less influence on both rare and abundant taxa.

## 4. Discussion

### 4.1. Potential impacts of hydrogeological properties and human activities on bacterial structure

Revealing the distribution pattern, environmental drivers, and assembly processes of bacterial communities is crucial for understanding the ecological function and health of karst groundwater (Gao et al., 2021; Tang et al., 2022). In this study, we conducted a comprehensive investigation of the distribution of groundwater bacterial communities based on 64 pristine groundwater samples across southwest China, one of the largest karst landforms in the world. The burial condition of groundwater primarily determines the hydrological connectivity and chemical characteristics of karst groundwater (Hill and Polyak, 2010; Larned, 2012). We observed a significant discrepancy in bacterial composition between phreatic and confined water, and the community similarity in phreatic water was significantly higher than that in confined water (*p* < 0.01). Confined aquifers are regarded as strictly anaerobic, oligotrophic, and isolated environments, providing ideal targets for the study of microbial evolution and environmental adaptation (Yan et al., 2021), while phreatic aquifers are usually closer to the Earth's surface, where groundwater is directly recharged by rainfall or snowmelt (Oliver et al., 2022). Thus, it was not surprising to observe the discrepancy in microbial structures between the two groundwater types. Meanwhile, the community similarity in phreatic water was higher than that in confined water, which could be attributed to the difference in hydrological connectivity between phreatic and confined water. Generally, phreatic aquifers in the karst region with characterized large voids, high flow velocities, and rapid infiltrations tend to strengthen microbial dispersal, while confined aquifers overlined by relatively impermeable rock or clay would reduce hydraulic connection and limit microbial diffusion (Flynn et al., 2012).

The specific hydraulic and hydrogeologic characteristics of karst aquifers render them highly vulnerable to pollution from human activities (Kacaroglu, 1999; Katsanou et al., 2013). Subject to the impacts of increasing pressure from heavy subterranean resource exploitation and anthropogenic contamination (Reberski et al., 2022), the potential ecological consequences (e.g., biodiversity loss) of karst aquifers have received extensive attention (Tang et al., 2022). In this study, we found that the potential functional groups associated with parasites and pathogens were much higher in karst groundwater than in other groundwater habitats (Liu et al., 2022), which were positively correlated with groundwater NO_3_-N content. Given that nitrate in groundwater is a typical anthropogenic pollutant and is widely used to indicate anthropogenic influences (Liu et al., 2015b; Opsahl et al., 2017), the prevalence of potential pathogen functional groups in karst groundwater could be closely linked to the footprints of anthropogenic activities (Stokdyk et al., 2020). Over the past decades, various antimicrobials, antibiotic-resistance genes, hormones, and microbial pathogens have been found widely distributed in groundwater (Manamsa et al., 2016) and are highly relevant to livestock and clinical waste (Hubbard et al., 2020). This study highlighted that bacteria would be a potential indicator for human impact evaluation of groundwater pathogen risk and drinking water safety. However, given the limitations of the potential functions predicted by FAPROTAX, the complementary confirmatory experiments combined with metabolome or transcriptome analyses would be further used to verify the feasibility of the bacterial indicator.

### 4.2. Ecological differentiation of rare and abundant taxa in karst groundwater

The bacterial community of karst groundwater in southwest China consists of a few abundant taxa and a high proportion of rare taxa, which is consistent with the most natural ecosystems (He et al., 2022; Zhao et al., 2022). As the most typical habitat generalist (Tully et al., 2018), Proteobacteria was observed to be the most abundant phylum in karst groundwater, with a higher proportion of abundant rather than rare subcommunities. These abundant taxa in karst groundwater may share some phenotypic traits or life history strategies to adapt to harsh subterranean habitats. For example, the genus Pseudomonas with the highest relative abundance (12.0%) in karst groundwater has been reported to have low nutritional requirements and can use various refractory organics (Vasquez-Ponce et al., 2018). On the contrary, the rare subcommunity harbored much higher phenotypic diversity and abundance of Patescibacteria and Chloroflexi than the abundant subcommunity. The newly defined superphylum Patescibacteria was prevalent in varying aquifer environments (Herrmann et al., 2019; Chaudhari et al., 2021; Ruiz-Gonzalez et al., 2022). In the absence of numerous biosynthetic capacities and stress response systems, it is confirmed that most Patescibacteria cannot live alone but be mutualists with other microbes, limiting their relative abundance in groundwater (Nelson and Stegen, 2015; Lemos et al., 2019). The majority of Chloroflexi members were classically phototrophic and were found to be abundant in surface water (Burganskaya et al., 2018; Gaisin et al., 2019). Although most Chloroflexi were rare taxa in groundwater, intensive study of these Chloroflexi members may provide a novel phototrophic mechanism in the deep biosphere (Zheng et al., 2022).

Revealing the underlying mechanisms of species coexistence within a specific ecological niche is of great importance for ecosystem restoration and environmental management (Yang Y. et al., 2022; Yang Z. et al., 2022). Based on bacterial co-occurrence networks in karst groundwater (Figure 6C), the positive correlations of mutualism, parasitism, or commensalism were prevalent among groundwater bacteria, whereas the negative correlations of competition for space and resources less occurred, indicating that bacterial co-occurrence based on cooperative interrelation was vital to community stability even in oligotrophic environments (Herren and McMahon, 2017). The node-level topological features of AT were significantly higher than those of RT, indicating that AT was more frequently central in the network. The key taxa of the abundant subcommunity mainly were module hubs, which were regarded as integral elements within distinct modules and may mediate important functions (Shi et al., 2020). The keystone species of RT were mainly identified as connectors, which contribute to community recovery under disturbance and might provide a buffer against environmental fluctuations (Yang Y. et al., 2022; Yang Z. et al., 2022). Previous studies found that rare taxa representing a substantial amount of ecological potential might achieve “rare-to-prevalent” dynamics over time as a response to disturbance events to maintain community stability and ecological function (Lynch and Neufeld, 2015; Nyirabuhoro et al., 2020). Generally, connectors are more conserved than module hubs (Guimera and Amaral, 2005). The keystone species of AT were more sensitive to various environments than those of the rare subcommunity, which supported the view that RT has a disproportionately large effect on ecological stability relative to its abundance (Shade et al., 2014; Jiao and Lu, 2020).

### 4.3. Higher environmental adaptation and stochasticity of rare than abundant taxa

Our results revealed that RT in karst groundwater was environmentally less constrained and showed wider environmental thresholds in response to most hydrochemical factors compared with AT. The results were consistent with those studies carried out in regions that suffered strong changes (He et al., 2022), but in contrast to most studies on surface soil, which tends to be rich in nutrients (Jiao and Lu, 2020). He et al. found that the stronger environmental adaptability of RT than AT during the reforestation ecological succession process was mainly associated with soil electrical conductivity (He et al., 2022). In general, rare taxa are regarded as a vital repository of specialists, while abundant taxa mainly comprise generalists (Xu et al., 2021). Specialists could be more competitive within a narrow niche breadth (Friedman et al., 2017), while generalists are more adaptable to habitat variations (Wang S. et al., 2019; Wang Y. et al., 2019). Karst aquifers in southeast China with anisotropic hydraulic connections would provide enough wide niche breadth for various specialists to survive (Opsahl et al., 2017), which increased the diversity of the rare subcommunity and may further enhance the important roles of rare taxa against environmental fluctuations (Yang Y. et al., 2022; Yang Z. et al., 2022). Therefore, the higher proportion of RT (specialists) would provide an important adaptability strategy for the bacterial community in extreme environments (e.g., karst groundwater), which is of great significance to broadening environmental threshold ranges and maintaining community stability.

Nowadays, there is still an ongoing debate about the relative contributions of deterministic and stochastic processes in the community assembly of rare and abundant taxa (Li et al., 2021; Yang Y. et al., 2022; Yang Z. et al., 2022). In this study, bacterial community assembly in karst groundwater was dominated by deterministic processes. More specifically, community assembly is mainly governed by variable selection (33.7%) rather than homogeneous selection (19.0%). Previous studies have demonstrated that variable selection (i.e., heterogeneous selection) caused by environmental heterogeneity would result in community divergence across localities and has been regarded as the most important ecological process of community assembly in natural habitats, especially in extreme environments (Wang et al., 2013; Evans et al., 2017). Meanwhile, we found that neutral processes had a significant impact on shaping the RT compared with the AT. Deterministic processes, especially homogeneous selection (35.6%), tended to be more important in shaping the subcommunity assembly of AT, while stochastic processes dominated by dispersal limitation (55.2%) and drift (20.3%) were the main assembly processes of rare subcommunities. Homogeneous selection could result in low compositional turnover (Zhou et al., 2013; Wang et al., 2020), which supported the result of much higher community similarity among abundant than rare subcommunities, whereas stochastic processes, such as ecological drift driven by stochastic demographic events, could result in dissimilar communities among localities even though sharing similar environmental conditions; thus, RT exhibited more randomness in phylogenetic clustering (Zhou et al., 2013; Stegen et al., 2015). Furthermore, higher dispersal limitations of RT could enhance the distance–decay relationship and result in high spatial species turnover (Zhou and Ning, 2017; Wang et al., 2020).

## 5. Conclusion

Our study provided a comprehensive investigation of the bacterial communities in karst groundwater in southwest China. Proteobacteria was the most abundant phylum in both AT and RT, while Patescibacteria and Chloroflexi flourished more in RT than AT. AT was more frequently central in the network and played important roles in maintaining bacterial community stability. Compared with AT, RT in karst groundwater exhibited stronger environmental adaptability and might contribute to community recovery under disturbance and provide a buffer against environmental fluctuations. Homogeneous selection belonging to deterministic processes dominated the abundant bacterial assembly, whereas dispersal limitation and drift belonging to stochastic processes governed the rare taxa. Meanwhile, we linked the high abundance of potential functional groups associated with parasites and pathogens in karst groundwater to frequent regional anthropogenic activities. This study significantly advances the knowledge of ecological differentiation and assembly processes of rare and abundant bacteria in typical karst aquifers and contributes to the microbial ecological prediction of ecosystem health and drinking water safety in karst regions.

## Data availability statement

The datasets presented in this study can be found in online repositories. The names of the repository/repositories and accession number(s) can be found below: NCBI - PRJNA692269.

## Author contributions

SZ and BL designed the research and wrote the manuscript. SZ performed the research with the help of BH, JZ, YW, XX, and JN. All authors contributed new ideas and participated in the interpretation of the findings. All authors contributed to the article and approved the submitted version.
