# Supplementary material for: Ecological differentiation and assembly processes of abundant and rare bacterial subcommunities in karst groundwater
Source: Front Microbiol. 2023 Jul 25;14:1111383. doi: 10.3389/fmicb.2023.1111383 (PMC10407230; doi:10.3389/fmicb.2023.1111383)
Supplement: Supplementary file 1 [file Table_1.docx]

Supplementary Material

Ecological differentiation and assembly processes of abundant and rare bacterial subcommunities in karst groundwater

**Sining Zhong^1,2,3^, Bowen Hou^4^, Jinzheng Zhang^1^,** **Yichu Wang^2,3^, Xuming Xu^2,3^, Bin Li ^2,3*^, Jinren Ni^2,3^**

^1^Fujian Agriculture and Forestry University, College of Resources and Environment, Fujian Provincial Key Laboratory of Soil Environment Health and Regulation, Fuzhou 350002, China

^2^College of Environmental Sciences and Engineering, Peking University; Key Laboratory of Water and Sediment Sciences, Ministry of Education, Beijing 100871, China

^3^State Environmental Protection Key Laboratory of All Material Fluxes in River Ecosystems, Peking University, Beijing 100871, China

^4^State Key Laboratory of Eco-hydraulics in Northwest Arid Region of China, Xi’an University of Technology, Xi’an 710048, China

*** Correspondence:** Bin Li, College of Environmental Sciences and Engineering, Peking University, Beijing 100871, P. R. China

E-mail: see-libin@pku.edu.cn

# Supplementary Figures


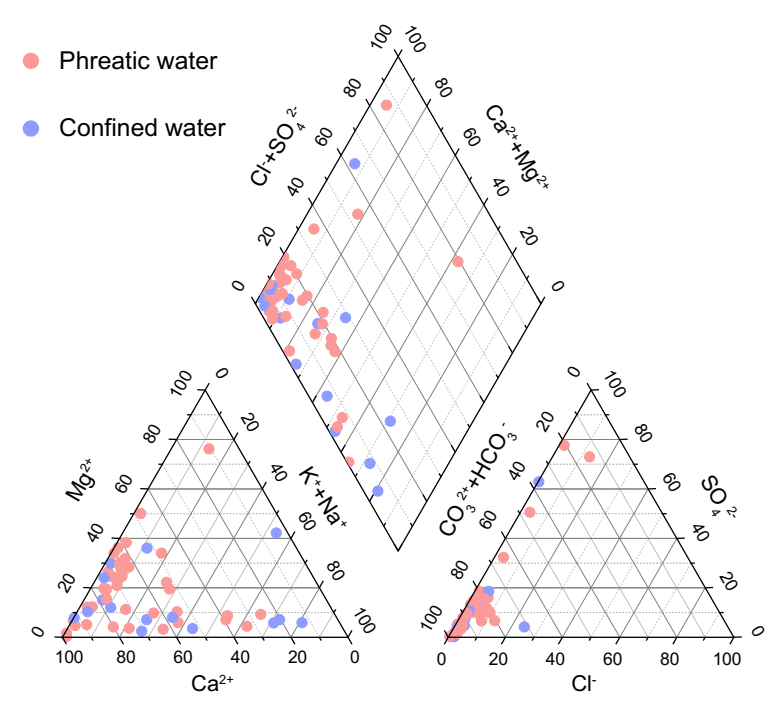


**Supplementary Fig. 1** Piper diagrams showing the hydrochemical characteristics of karst groundwater in phreatic (red circle) and confined (blue circle) water.


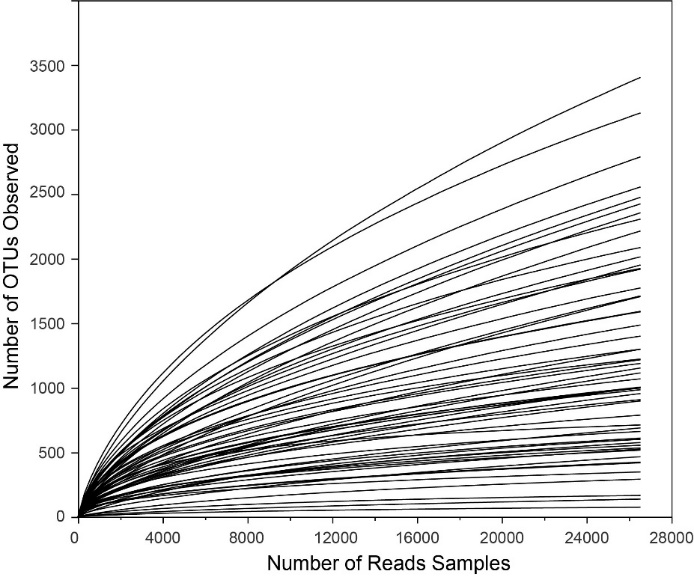


**Supplementary Figure 2** Rarefaction curves of bacterial richness in each sample of karst groundwater in Southwest China.


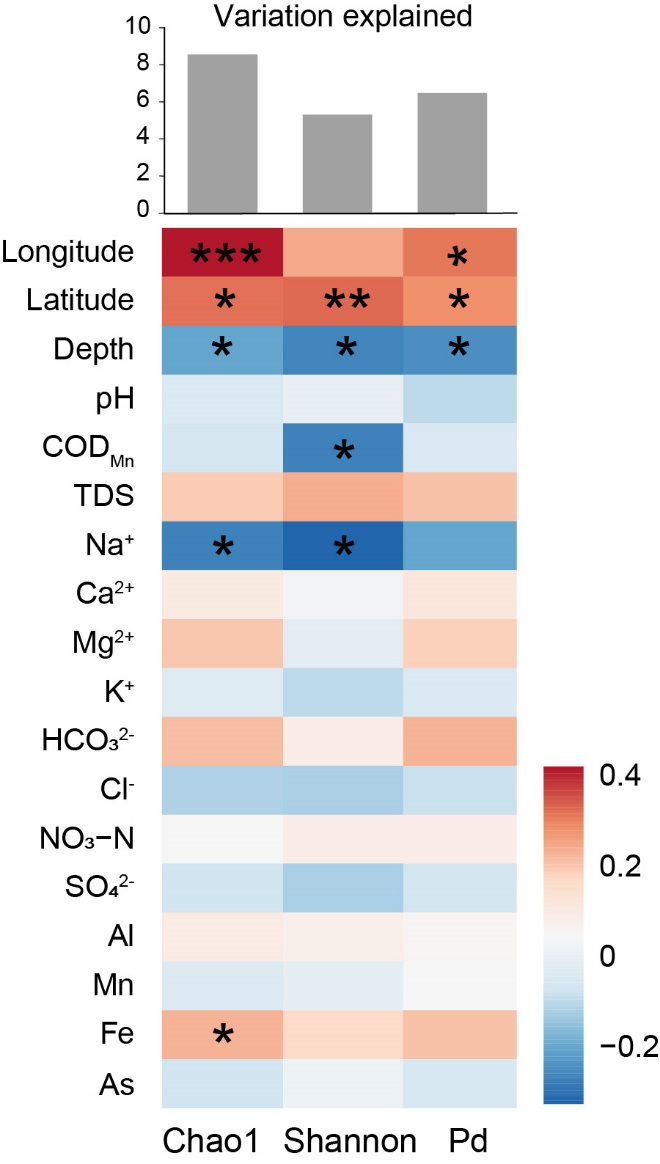


**Supplementary Figure 3** The mainly environmental factors for explaining the bacterial diversity in karst groundwater by the Random Forest model and Spearman correlations. Bacterial diversity is represented by Chao1, Shannon diversity, and Phylogenetic diversity (pd). The heatmap with red and blue color show positive and negative correlations between environmental factors and diversity index, respectively. Asterisks denote significant Pearson correlations. **0.001 *< p <* 0.01, and *0.01 *< p <* 0.05.


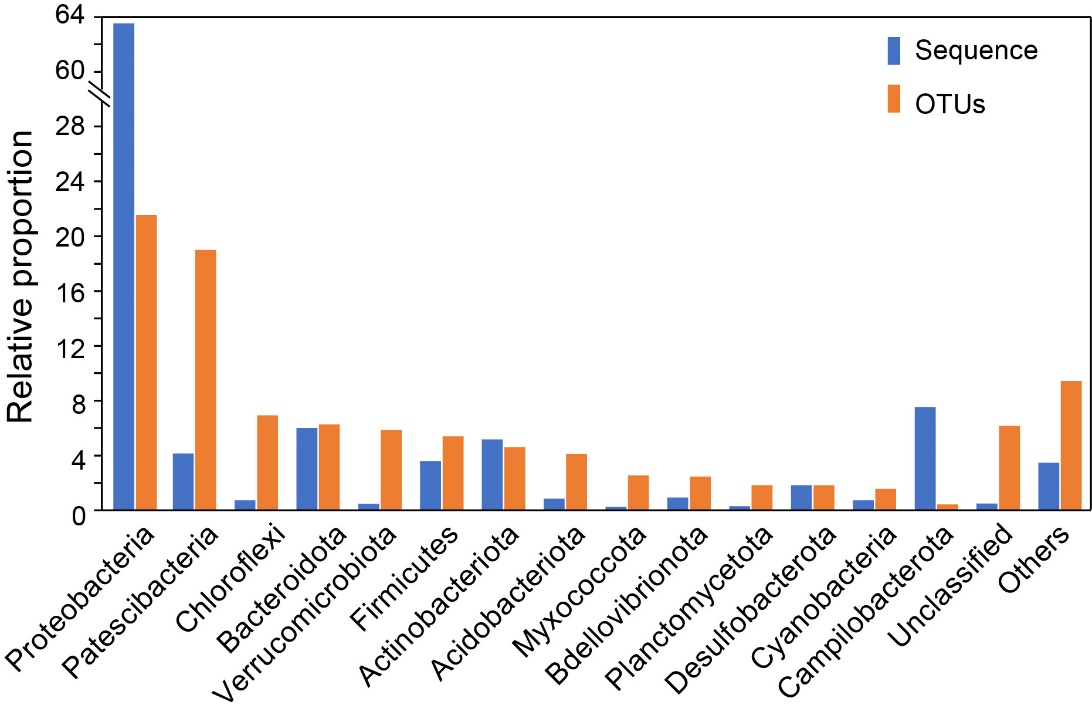


**Supplementary Figure 4** Relative proportion of OTUs and total sequences for the major phyla in karst groundwater. Only the phyla with mean relative proportion of > 1% are shown.


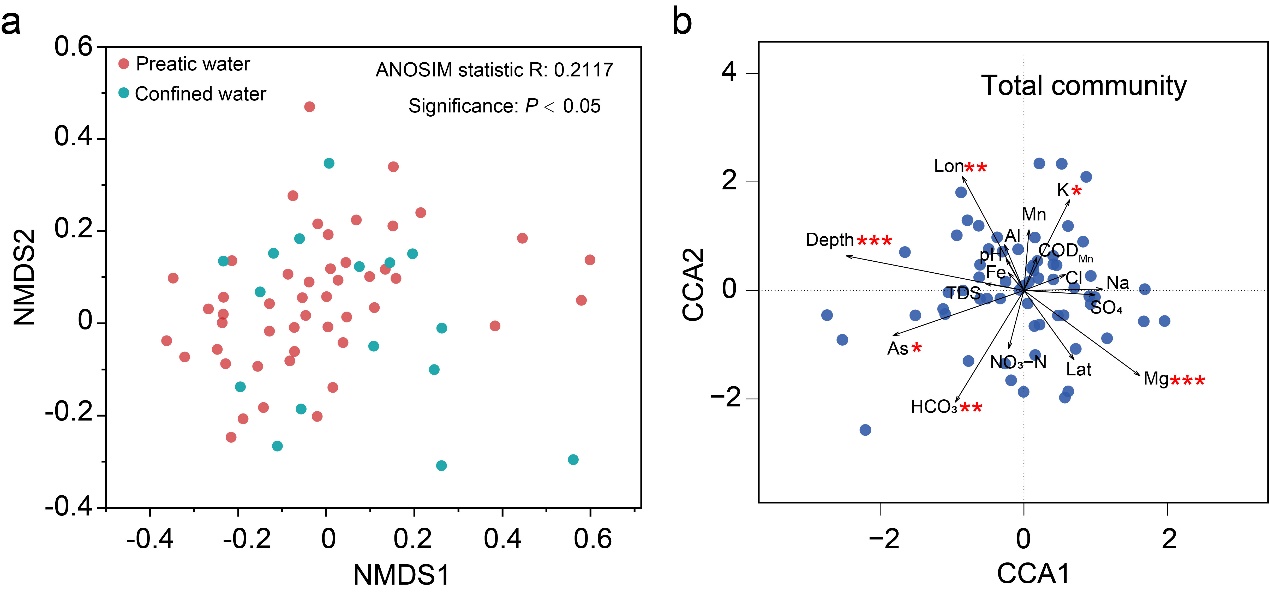


**Supplementary Figure 5** (a) NMDS showing bacterial compositional differences between phreatic and confined water. The ANOSIM *R* and *p* values are provided. (b) CCA test showing the relationships between environmental factors and bacterial compositions of karst groundwater in Southwest China. ***0.001 > *p*, **0.001 < *p* < 0.01, and *0.01 < *p* < 0.05.


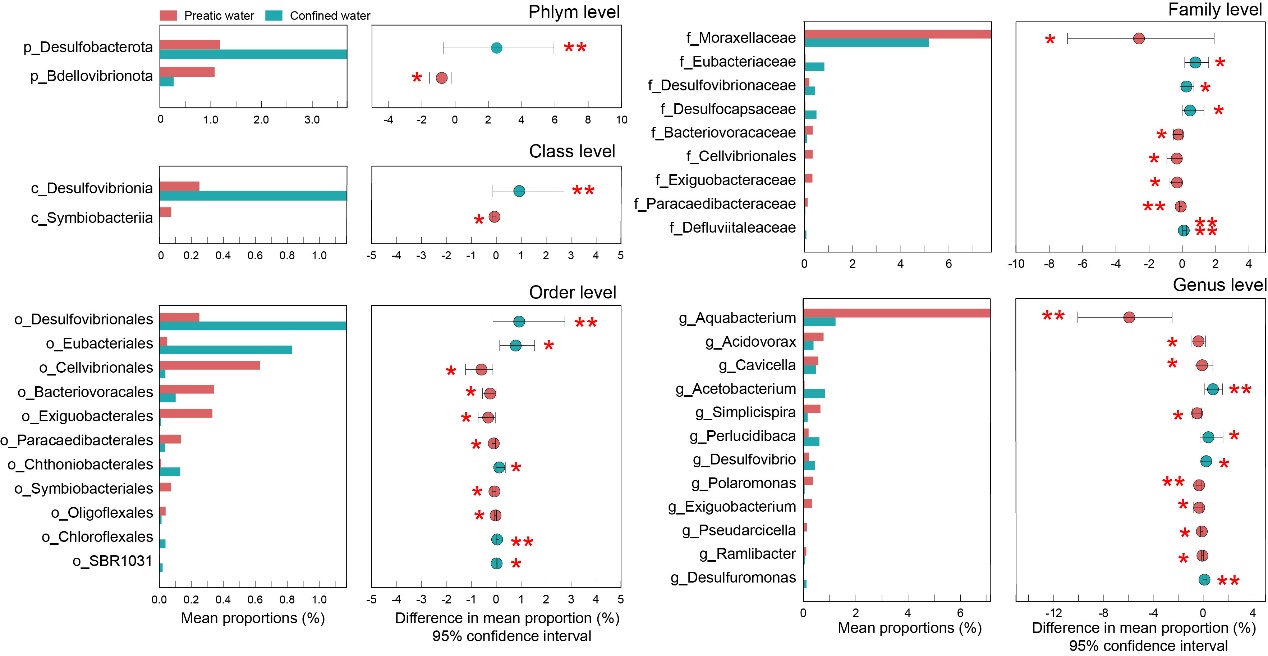


**Supplementary Figure 6** Environmental preferences of mainly taxa between phreatic and confined water at phylum, class, order, and genus levels. **0.001 < *p* < 0.01, and *0.01 < *p* < 0.05.


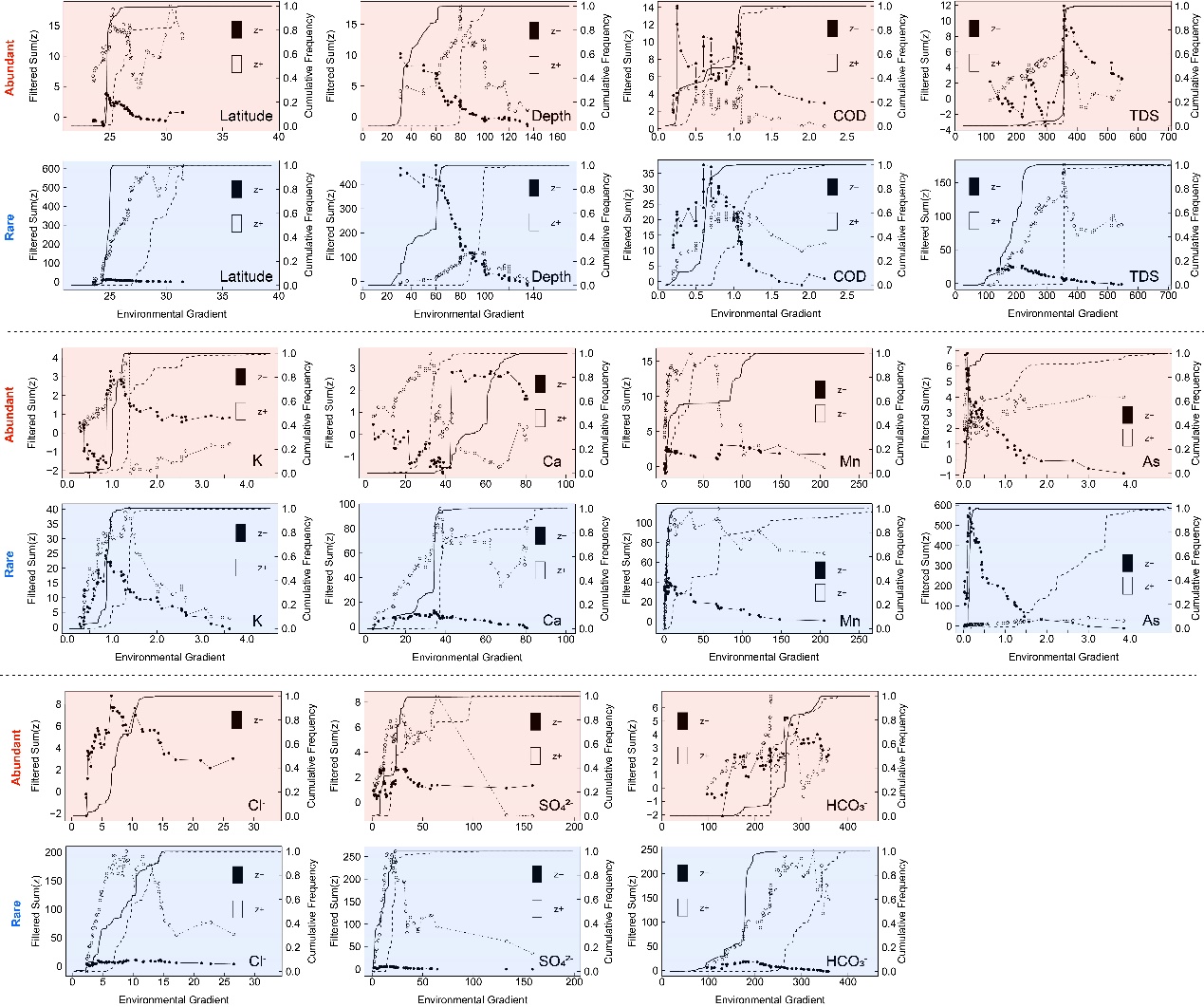


**Supplementary Figure 7** Occurrence thresholds of abundant and rare bacteria in karst groundwater with respect to mainly environmental variables with significant effects on community composition and structure. The z-scores of all community members are shown.


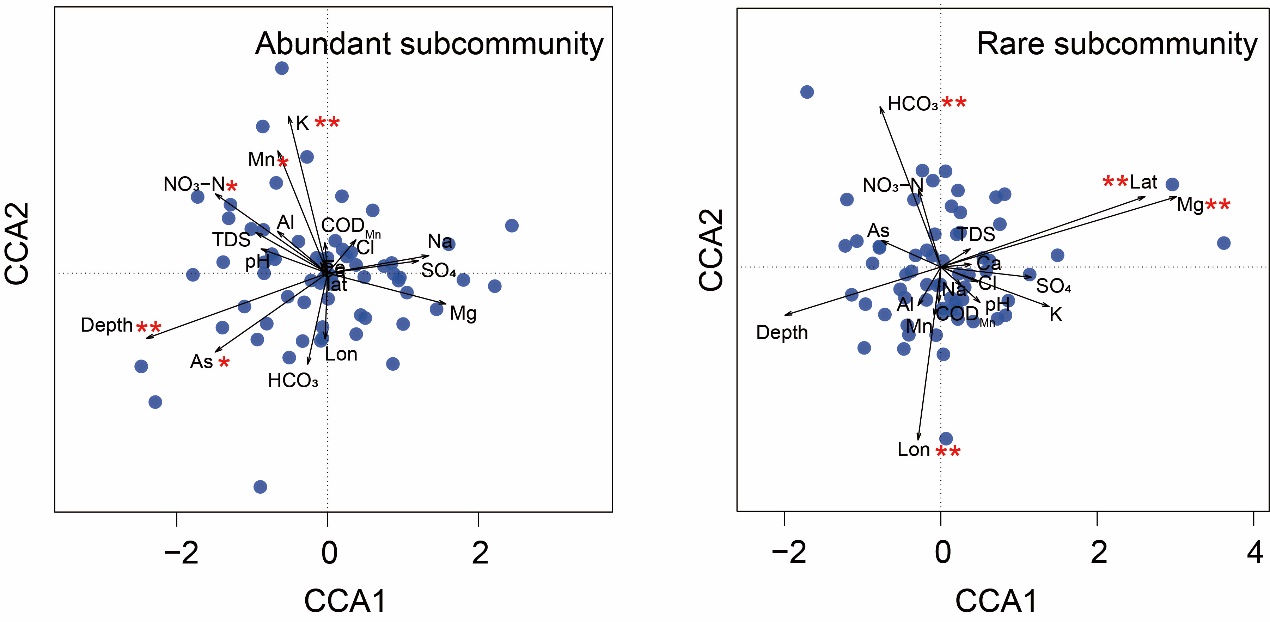


**Supplementary Figure 8** CCA test showing the relationships between environmental factors and bacterial structures of abundant and rare subcommunity in karst groundwater. ***0.001 > *p*, **0.001 < *p* < 0.01, and *0.01 < *p* < 0.05.


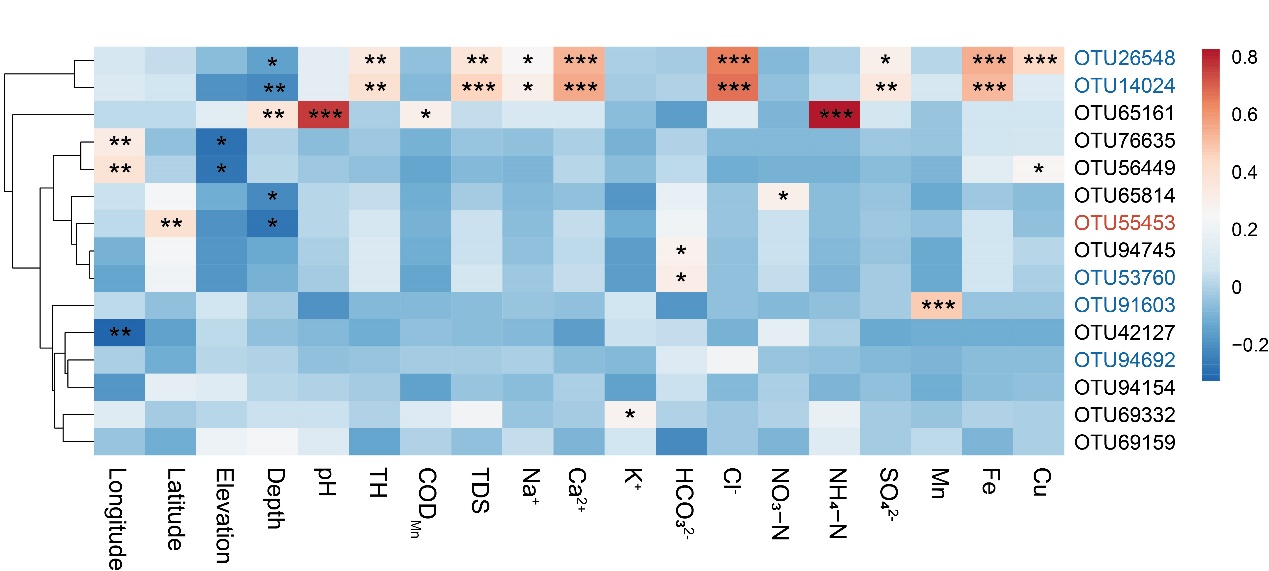


**Supplementary Figure** **9** Heatmap showing the influence of groundwater hydrochemical factors on the abundance of module hubs based on Spearman’s correlation coefficients. All the asterisks denote the significance of statistical tests: ***0.001 > *p*, **0.001 < *p* < 0.01, and *0.01 < *p* < 0.05.

# Supplementary Tables

**Supplementary Table 1** The proportion of karst groundwater in southwest China for varying water quality levels according to the Standard for Groundwater Quality of China (GB/T 14848-2017).

| **Indices** | **Level I (%)** | **Level II (%)** | **Level III (%)** | **Level IV (%)** | **Level V (%)** |
| --- | --- | --- | --- | --- | --- |
| NO_3_^-^ | 14.5 | 21.8 | 41.8 | 14.6 | 7.3 |
| Mn | 73.4 | 0.0 | 9.4 | 7.8 | 9.4 |
| F^-^ | 83.0 | 0.0 | 0.0 | 13.2 | 3.8 |
| NH_4_^+^ | 0.0 | 46.4 | 39.3 | 7.1 | 7.2 |
| Fe | 60.9 | 21.9 | 7.8 | 9.4 | 0.0 |
| Hardness | 25.0 | 46.4 | 21.4 | 3.6 | 3.6 |
| COD_Mn_ | 67.9 | 21.4 | 5.4 | 5.4 | 0.0 |
| Na^+^ | 95.3 | 0.0 | 3.1 | 0.0 | 1.6 |

**Supplementary Table 2** Statistical characteristic of bacterial diversity index in karst groundwater of southwestern China.

| Estimators | Mean | Standard deviation | Coefficient of variation | Kolmogorov-Smirnov test |
| --- | --- | --- | --- | --- |
| Shannon | 3.84 | 1.26 | 0.33 | 0.08 (Normal distribution) |
| Chao | 2228.74 | 1473.02 | 0.66 | 0.20 (Normal distribution) |
| Pd | 136.44 | 78.08 | 0.57 | 0.20 (Normal distribution) |

**Supplementary Table 3** The identified module hubs of co-occurrence network and their main node-level topological metrics.

| OTU name | group | Phylum | Zi | Pi | Degree | Closeness | Betweenness |
| --- | --- | --- | --- | --- | --- | --- | --- |
| OTU94692 | Abundant | Proteobacteria | 3.14 | 0.30 | 12 | 0.18 | 38558.4 |
| OTU91603 | Abundant | Proteobacteria | 2.58 | 0.08 | 24 | 0.14 | 6921.3 |
| OTU14024 | Abundant | Proteobacteria | 2.89 | 0.14 | 27 | 0.15 | 4913.3 |
| OTU53760 | Abundant | Proteobacteria | 2.91 | 0.07 | 27 | 0.20 | 8984. 7 |
| OTU26548 | Abundant | Actinobacteriota | 3.66 | 0.06 | 31 | 0.15 | 5844.0 |
| OTU65161 | Intermediate | Proteobacteria | 2.74 | 0.08 | 25 | 0.15 | 4880.1 |
| OTU69159 | Intermediate | Proteobacteria | 2.89 | 0.08 | 26 | 0.14 | 2770.3 |
| OTU94154 | Intermediate | Proteobacteria | 2.60 | 0.15 | 26 | 0.22 | 58392.7 |
| OTU65814 | Intermediate | Methylomirabilota | 2.54 | 0.09 | 23 | 0.16 | 1270.0 |
| OTU76635 | Intermediate | Bacteroidota | 2.57 | 0.15 | 13 | 0.12 | 7968.0 |
| OTU94745 | Intermediate | Proteobacteria | 2.60 | 0.08 | 25 | 0.20 | 7935.7 |
| OTU42127 | Intermediate | Proteobacteria | 3.14 | 0.17 | 11 | 0.16 | 3759.1 |
| OTU56449 | Intermediate | Actinobacteriota | 3.18 | 0.13 | 15 | 0.12 | 3202.8 |
| OTU69332 | Intermediate | Firmicutes | 3.49 | 0.12 | 16 | 0.15 | 13860.2 |
| OTU55453 | Rare | Acidobacteriota | 2.60 | 0.36 | 5 | 0.15 | 2544.6 |
